# Structure and selected properties of Al–Cr–Fe alloys with the presence of structurally complex alloy phases

**DOI:** 10.1038/s41598-022-17870-0

**Published:** 2022-08-20

**Authors:** K. Młynarek-Żak, W. Pakieła, D. Łukowiec, A. Bajorek, P. Gębara, A. Szakál, I. Dhiman, R. Babilas

**Affiliations:** 1 Department of Engineering Materials and Biomaterials, Silesian University of Technology, Konarskiego 18a, 44-100 Gliwice, Poland; 2 A. Chełkowski Institute of Physics, University of Silesia in Katowice, 75 Pułku Piechoty 1, 41-500 Chorzów, Poland; 3 Department of Physics, Częstochowa University of Technology, Armii Krajowej 19, 42-200 Częstochowa, Poland; 4 Budapest Neutron Centre, Konkoly-Thege Miklos st. 29-33, Budapest, 1121 Hungary

**Keywords:** Characterization and analytical techniques, Materials science, Mechanical properties, Metals and alloys

## Abstract

The aim of the study was to supplement the data on the Al_65_Cr_20_Fe_15_ alloy with binary phase structure and the Al_71_Cr_24_Fe_5_ alloy with multiphase structure prepared with two different cooling rates from the liquid state. The presence of the structurally complex Al_65_Cr_27_Fe_8_ phase was confirmed by neutron diffraction, scanning electron microscopy with the analysis of chemical composition and transmission electron microscopy. Additionally, the Al_8_Cr_5_ phase with γ-brass structure was identified for Al_71_Cr_24_Fe_5_ alloy in both cooling rates from the liquid state. Due to the interesting features of structurally complex alloys, the wear resistance, magnetic properties, and corrosion products after performing electrochemical tests were examined. Based on pin-on-disc measurements, a lower friction coefficient was observed for the Al_65_Cr_20_Fe_15_ alloy (µ ≈ 0.55) compared to the Al_71_Cr_24_Fe_5_ multiphase alloy (µ ≈ 0.6). The average hardness of the binary phase Al_65_Cr_20_Fe_5_ alloy (HV_0.1_ = 917 ± 30) was higher compared to the multiphase Al_71_Cr_24_Fe_5_ alloy (HV_0.1_ = 728 ± 34) and the single phase Al–Cr–Fe alloys described in the literature. Moreover, the beneficial effect of rapid solidification on hardness was demonstrated. The alloys Al_65_Cr_20_Fe_15_ and Al_71_Cr_24_Fe_5_ showed paramagnetic behavior, however rapidly solidified Al_71_Cr_24_Fe_5_ alloy indicated an increase of magnetic properties. The studied alloys were characterized by the presence of passive layers after electrochemical tests. A higher amount of oxides on the surface of the Al_71_Cr_24_Fe_5_ alloy was recorded due to the positive effect of chromium on the stabilization of the passive layer.

## Introduction

Complex metallic alloys (CMAs) are intermetallic crystalline compounds. CMAs are composed of structurally complex alloy phases (SCAPs)^1^. They are characterized by large unit cells that can be made up of thousands of atoms. Crystals that contain several dozen atoms in their cell^2^, quasicrystals, and their approximants^3^ are considered SCAP-type structures. Complex metallic alloys indicate interesting physicochemical properties, such as high hardness, low friction coefficient, and good corrosion resistance^4,5^. Moreover, SCAPs free from structure defects can be characterized by a high degree of magnetic order^6^. The set of unique characteristics of alloys with the structurally complex structure results from differences in the transport of electrons and phonons due to the different atomic structures of classical crystal lattices^2,7^. The main limitations for the development of this group of materials is the manufacturing of structurally complex single-phase alloys and the computational and theoretical resources for their description^6^. Based on physicochemical properties, complex metallic alloys have potential applications as thermoelectric, catalytic, and structural materials (among others, in high-load satellite parts)^3,5,6^. CMAs could be applied in composites or as coating materials due to the reduced friction coefficient^4,5,8^.

The Al–Cr–Fe, Al–Cu–Fe and Al–Cu–Fe–Cr alloys were classified as CMAs due to the presence of a structurally complex alloy phases^4^. The occurrence of γ-brass phases was often observed during the preparation of quasicrystals and their approximants in Al–Cr^9,10^, Al–Cr–Fe^2,4,8,11–14^, Al–Cu^15,16^, and Al–Cu–Cr^17,18^ chemical compositions^12^. Dong^9^ stated that the γ-brass phases are approximations to quasicrystals. Similarly, Veys et al.^19^ indicated that the phase of Al_65_Cr_27_Fe_8_ is a CMA compound with γ-brass structure that can be considered as an approximant of quasicrystalline icosahedral and decagonal phases. In other publications^4,11^, the alloys Al_64.2_Cr_27.2_Fe_8.1_ and Al_66.9_Cu_11.6_Fe_11.6_Cr_10.6_ of structurally complex alloys were produced by hot sintering powders in the form of rolls with a diameter of 20 mm and then subjected to heat treatment. Based on the X-ray diffraction analysis, the Al_8_Cr_5_ phase was identified for the Al_64.2_Cr_27.2_Fe_8.1_ alloy and Al_6.5_Cr_0.5_Cu_2_Fe phase for the Al_66.9_Cu_11.6_Fe_11.6_Cr_10.6_ alloy. The authors^11^ concluded that the Al_8_Cr_5_ phase (γ-brass) is isostructural with the Al_65_Cr_27_Fe_8_ phase.

The purpose of the work was to provide detailed structural studies of the Al_65_Cr_20_Fe_15_ and Al_71_Cr_24_Fe_5_ alloys produced with two different cooling rates from the liquid state. In addition to the earlier work^20^, these alloys have not been described in terms of structure so far. Moreover, there is still a few experimental data that confirm the interesting properties of Al–Cr–Fe alloys with the CMAs, especially with binary and multiphase structure^4,5^. The results of the selected properties such as wear resistance, hardness, magnetic behavior, and chemical composition of the surface after corrosion were analyzed.

## Materials and methods

Chemical elements of Al, Cr, and Fe with a purity of 99.99% were melted in an induction furnace with appropriate atomic fractions (Al_71_Cr_24_Fe_5_ and Al_65_Cr_20_Fe_15_ at.%) in a protective argon atmosphere in corundum crucibles (Φ = 30 mm, H = 45 mm) and then Ar-cooled. Ingots produced with a weight of 50 g were remelted and cast with an increased cooling rate from a liquid state under pressure (high-pressure die casting method with a cooling rate ~ 10^3^ K/s) to a water-cooled copper mold (90 × 80 × 45 mm) in the form of plates (30 × 10 × 1 mm). X-ray diffraction, light microscopy observations, Mössbauer spectroscopy, differential scanning calorimetry, and electrochemical measurements were described for these alloys in an earlier publication^20^.

Neutron diffraction studies were performed on the MTEST neutron powder diffractometer at the Budapest Neutron Center. The Cu(111) monochromator was used which selected neutrons with a wavelength of λ = 0.1446 nm. The measured 2θ range was between 10° and 140°. This setup allowed a sufficient q-range and resolution for the identification of different phases present in the samples.

Observations of an ingot structure were made using scanning electron microscopy in backscattered electron (BSE) mode (Supra 35, Carl Zeiss) with EDX analysis to identify maps with the chemical composition of phases.

High resolution transmission electron microscopy (HRTEM) was used to determine electron diffraction from the selected area (SAED), structure, and morphology using S/TEM TITAN 80–300. Samples for HRTEM observations were powdered.

Coercive force (*H*_c_) and saturation magnetization (*M*_s_) were determined from changes of magnetization as a function of the magnetic fields up to 10 kOe. Magnetic properties were recorded using a LakeShore 7307 vibrating sample magnetometer.

Tribological tests were performed using the pin-on-disc method using CSM Instruments. The experiments were carried out on cylindrical ingots with a radius of 26 mm and a height of 3 mm. The radius of the wear track was 8 mm. A ball made of 100C6 steel with a diameter of 6 mm was used as a counter-sample. The linear speed was 0.01 m/s and a load of 10 N was applied. Observations of the wear tracks, together with measurements of their width after tribological tests, were carried out by scanning electron microscope (Supra 35, Carl Zeiss). Hardness tests were performed using a Future Tech FM-700 Vickers hardness testing instrument with a load of 100 g for 15 s.

The corrosion products on the surface of the Al_65_Cr_20_Fe_15_ and Al_71_Cr_24_Fe_5_ samples in the form of plates after corrosion tests in 3.5% NaCl solution at 25 °C were determined by X-ray photoelectron spectroscopy (XPS). Depth profile mode (DP-XPS) using a Physical Electronics (PHI 5700/660) spectrometer working under an ultra-high vacuum (10^−9^ Torr) in UHV cluster and a monochromatic Al Kα X-ray source (1486.6 eV) was used. Both tested samples were initially kept pre-chamber held under vacuum (10^−8^ Torr) for at least 1 h, next transferred to the measurement chamber and analyzed. The survey spectra were measured with a pass energy of 187.85 eV. Depth profile (DP-XPS) analysis was carried out using a focused 1.5 kV Ar^+^ beam for 15 min, sputtering in intervals between measurements. Core-level lines collected in the DP-XPS analysis were measured with a pass energy of 23.5 eV. All obtained XPS data was analyzed using MultiPak 9.7 software, which contains an internal reference database and compared to the NIST XPS database.

### Ethical approval

This article does not contain any studies with human participants or animals performed by any of the authors.

## Results and discussion

**Figure 1:**
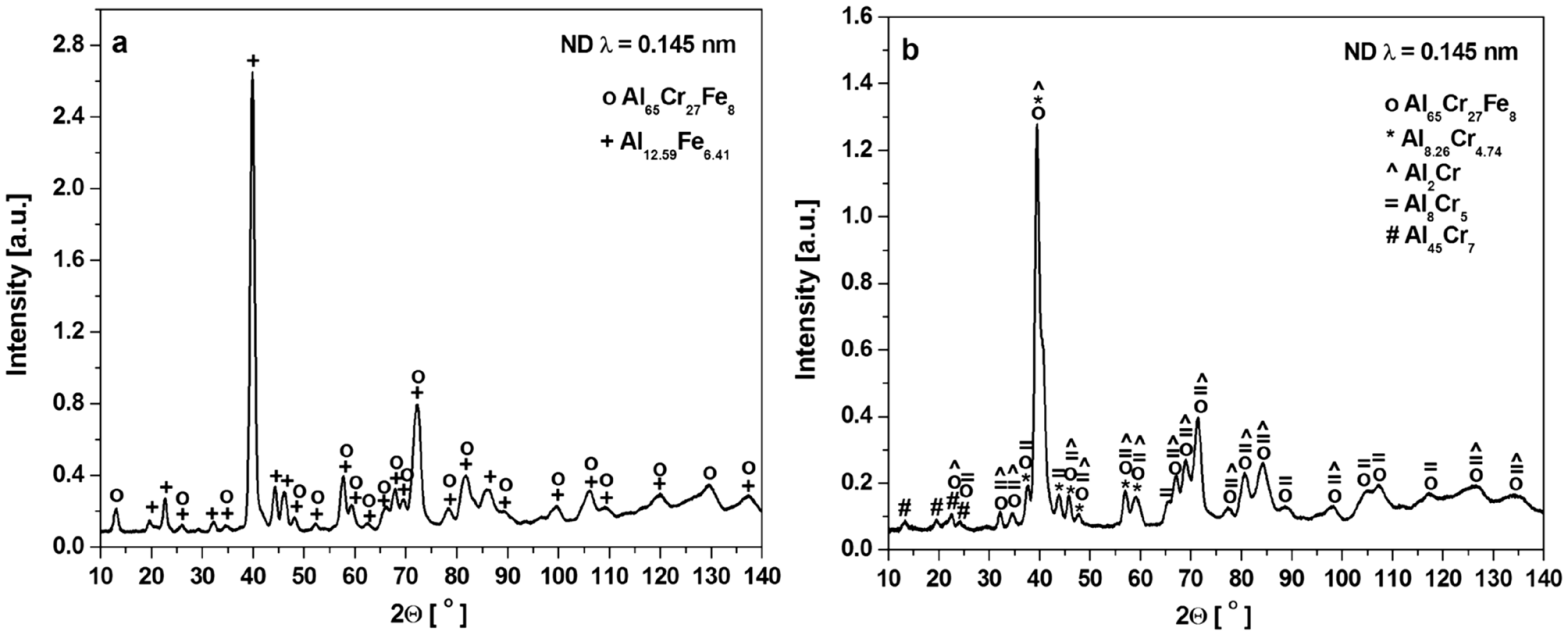
ND patterns of Al_65_Cr_20_Fe_15_ (**a**) and Al_71_Cr_24_Fe_5_ (**b**) alloys in a form of ingot.

**Figure 2:**
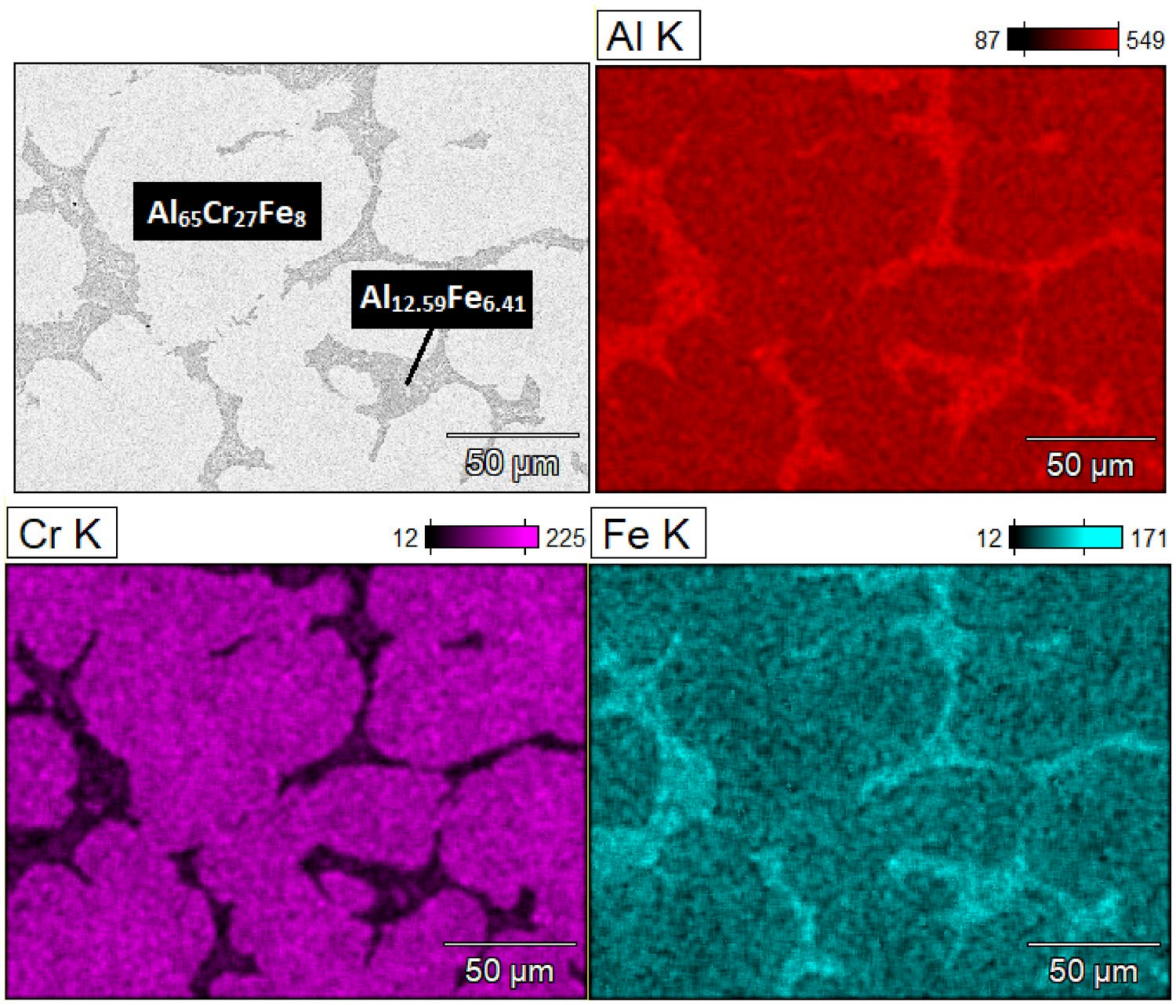
EDX maps of Al_65_Cr_20_Fe_15_ alloy in a form of ingot.

**Figure 3:**
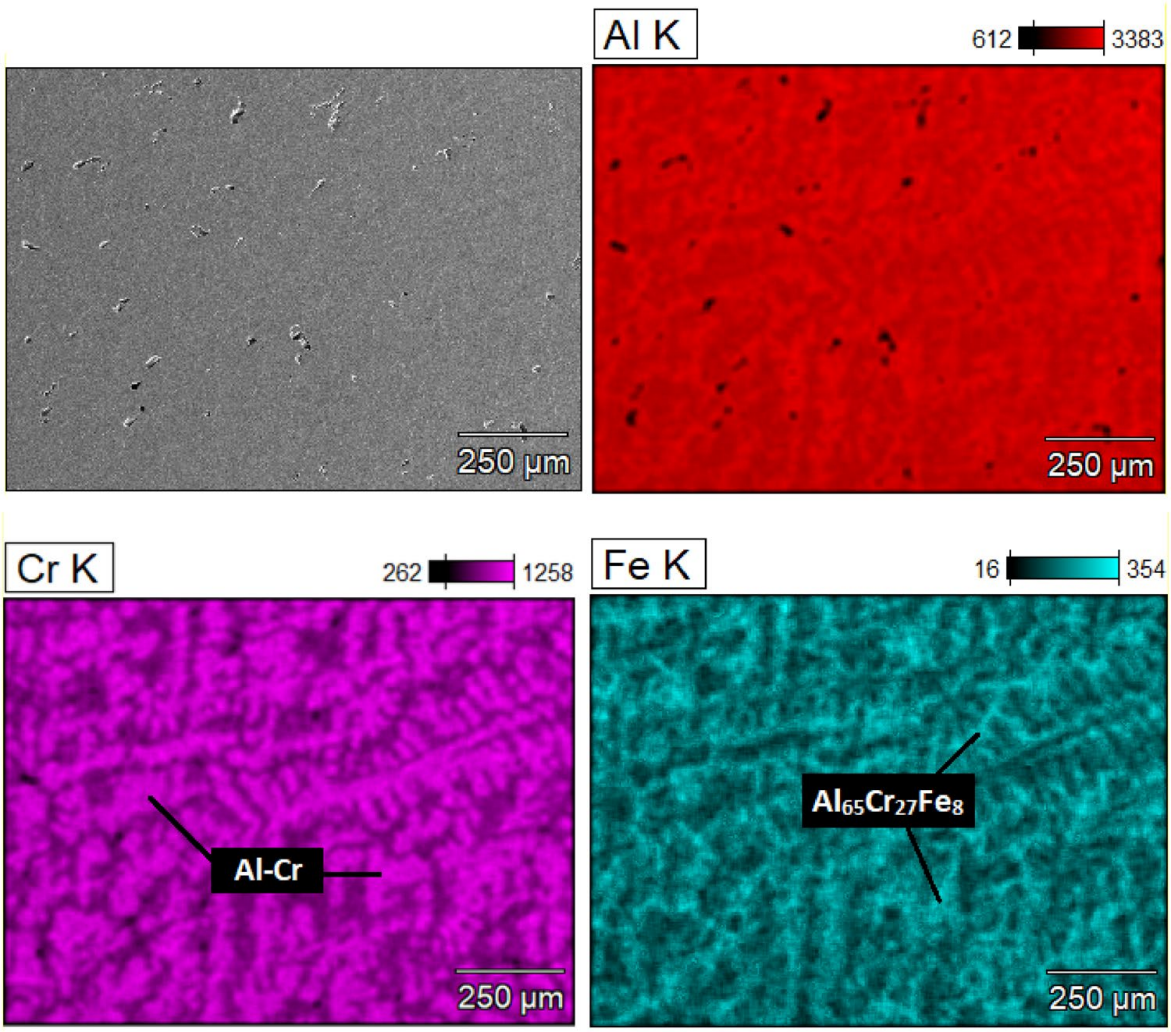
EDX maps of Al_71_Cr_24_Fe_5_ alloy in a form of ingot.

Based on the phase analysis provided by the XRD method presented in^20^, the Al_65_Cr_20_Fe_15_ alloy was characterized by a binary phase structure (ingot: Al_65_Cr_27_Fe_8_ (SCAP) + Al_12.59_Fe_6.41_, plate: Al_65_Cr_27_Fe_8_ (SCAP) + Al_5.6_Fe_2_), and the Al_71_Cr_24_Fe_5_ alloy, a multiphase (ingot: Cr + Al_65_Cr_27_Fe_8_ (SCAP) + Al_8.26_Cr_4.74_ + Al_2_Cr + Fe_2_CrAl + Al_8_Cr_5_ + Al_45_Cr_7_, plate: Cr + Al_65_Cr_27_Fe_8_ (SCAP) + Al_8.26_Cr_4.74_ + Al_2_Cr + Fe_2_CrAl + Al_8_Cr_5_ + Al_45_Cr_7_). The presence of the identified phases in the structure for the ingots was confirmed by the analysis of the neutron diffractograms in Fig. 1. The presence of the Fe_2_CrAl and Cr phases was excluded for Al_71_Cr_24_Fe_5_ due to the small number of matched reflections. Furthermore, observations were carried out using scanning electron microscopy in BSE mode with the EDX analysis presented in Fig. 2 for Al_65_Cr_20_Fe_15_ and Fig. 3 for Al_71_Cr_24_Fe_5_ alloys. SEM observations confirmed the presence of two phases in the structure of the Al_65_Cr_20_Fe_5_ alloy. The presented EDX maps allow us to suppose that the alloy matrix was constituted by the Al_65_Cr_27_Fe_8_ (SCAP) phase, while the role of reinforcing phase was played by Al_12.59_Fe_6.41_. Identification of individual phases of the Al_71_Cr_24_Fe_5_ alloy was difficult due to the presence of many phases. However, iron-rich areas were identified suggesting the presence of the Al_65_Cr_20_Fe_8_ (SCAP) phase. The Al–Cr phases formed a dendritic structure.

**Figure 4:**
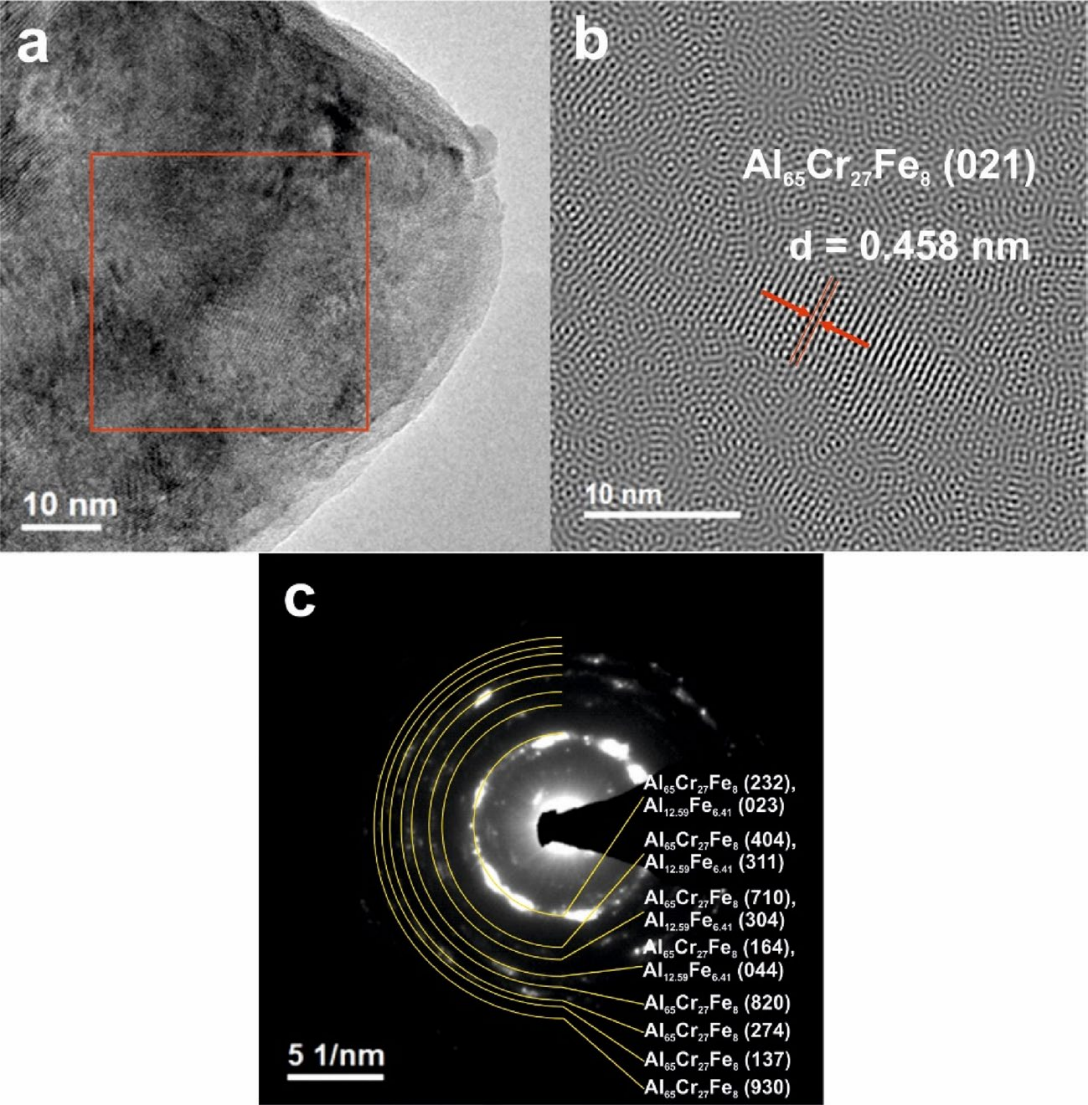
HRTEM image (**a**), IFT image (**b**) and SAED patterns (**c**) of Al_65_Cr_20_Fe_15_ plate.

**Figure 5:**
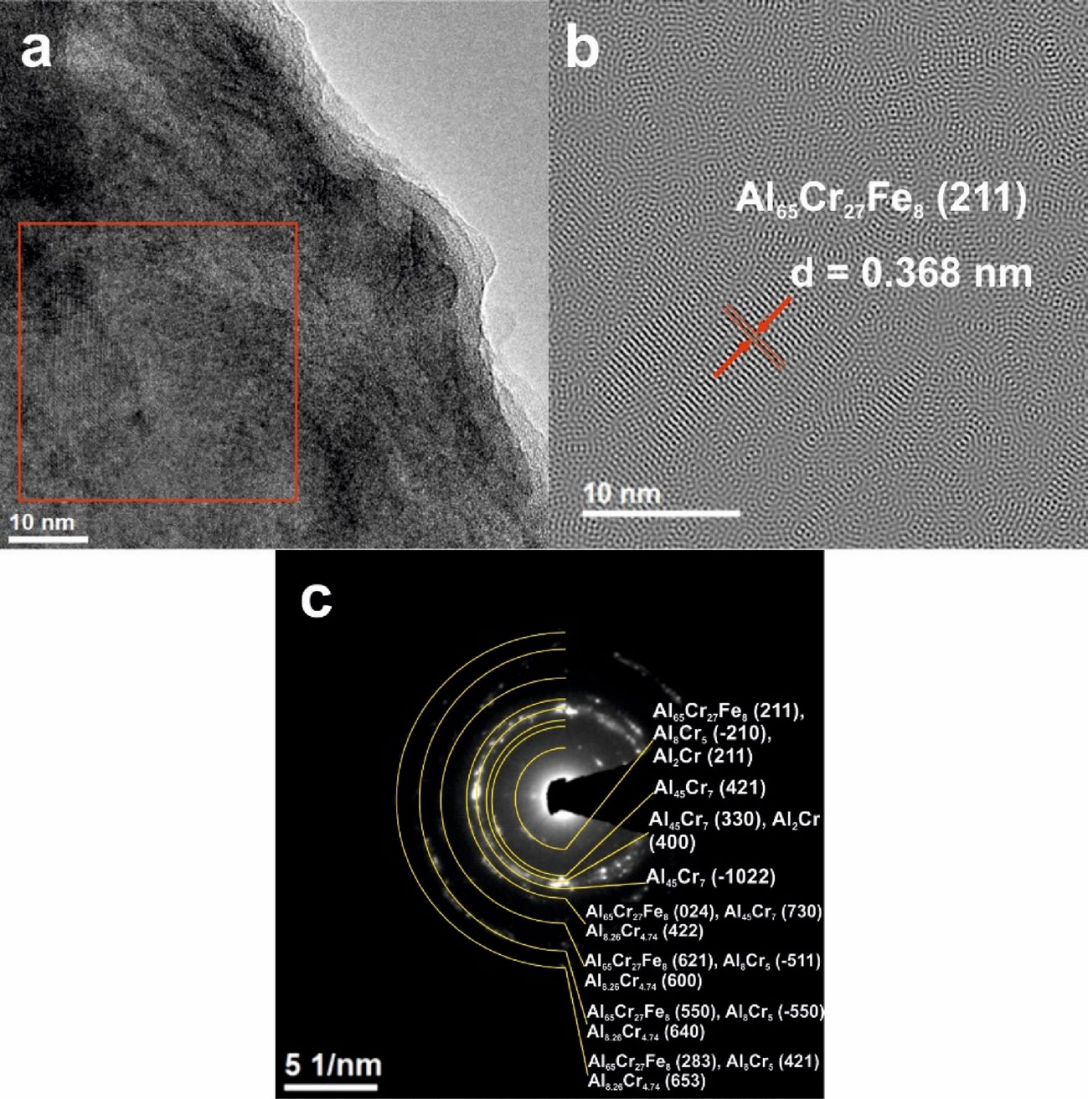
HRTEM image (**a**), IFT image (**b**) and SAED patterns (**c**) of Al_71_Cr_24_Fe_5_ plate.

In addition, studies with the use of transmission electron microscopy, presented in Figs. 4 and 5, were carried out. The presence of two phases in the structure of the Al_65_Cr_20_Fe_15_ alloy and multiple phases in Al_71_Cr_24_Fe_5_ was confirmed by selected area electron diffraction (SAED) patterns from the areas of Figs. 4a and 5a. TEM investigations were performed for samples in a form of plate. SAED analysis confirmed the ND results. The Inverse Fourier Transform (IFT) images also show areas with an ordered structure of atoms, characteristic of crystalline structures. The interplanar spacings in the marked crystalline areas were *d* = 0.458 nm (Fig. 4b) and *d* = 0.368 nm (Fig. 5b). The revealed values of the *d* spacings were found to closely with the interplanar spacings of the Al_65_Cr_27_Fe_8_ phase.

Al–Cr–Fe alloys were described in the literature primarily in terms of the formation of structurally complex phases. Ura-Binczyk et al.^4,11^ studied a polycrystalline alloy of Al_64.2_Cr_27.2_Fe_8.1_, which was produced by hot press sintering of the intermetallic powders and heat treated. The authors^11^ pointed out that γ-Al_8_Cr_5_ phase identified by XRD is isostructural with Al_65_Cr_27_Fe_5_. In this study, the Al_65_Cr_27_Fe_6_ phase had lattice parameters a = b = 12.6963 and c = 7.9211 and the angles between them α = β = 90° and γ = 120° in the hexagonal notation, which is consistent with the data reported in the articles^11,16^. According to report presented by Veys et al.^19^ the phase of Al_65_Cr_27_Fe_8_ has γ-brass structure and is isostructural to cubic Al_9_Cr_4_ (with lattice parameters a = 9.4 Å).

In this work, for the multiphase Al_71_Cr_24_Fe_5_ alloy, the Al_8_Cr_5_ phase was also identified for both cooling rates. The lattice parameters of which a = b = c = 7.8050 and α = β = γ = 109.127° correspond to the α-Al_8_Cr_5_ phase, which corresponds to the rhombohedral system. The parameters of the unitary lattice cell are consistent with the description^12^. According to^10,12,21^, the Al_8_Cr_5_ phase with a rhombohedral structure has a γ-brass structure.

Additionally, the results were compared with a study in which Al–Cr–Fe based alloys were cast^13^. Two phases were marked in the SEM image for the Al_66_Fe_22_Cr_12_ alloy: α-Al_8_Cr_5_ and Al_5_Fe_2_. The microstructure is similar to that shown in Fig. 2 for Al_65_Cr_20_Fe_15_^13^.

**Figure 6:**
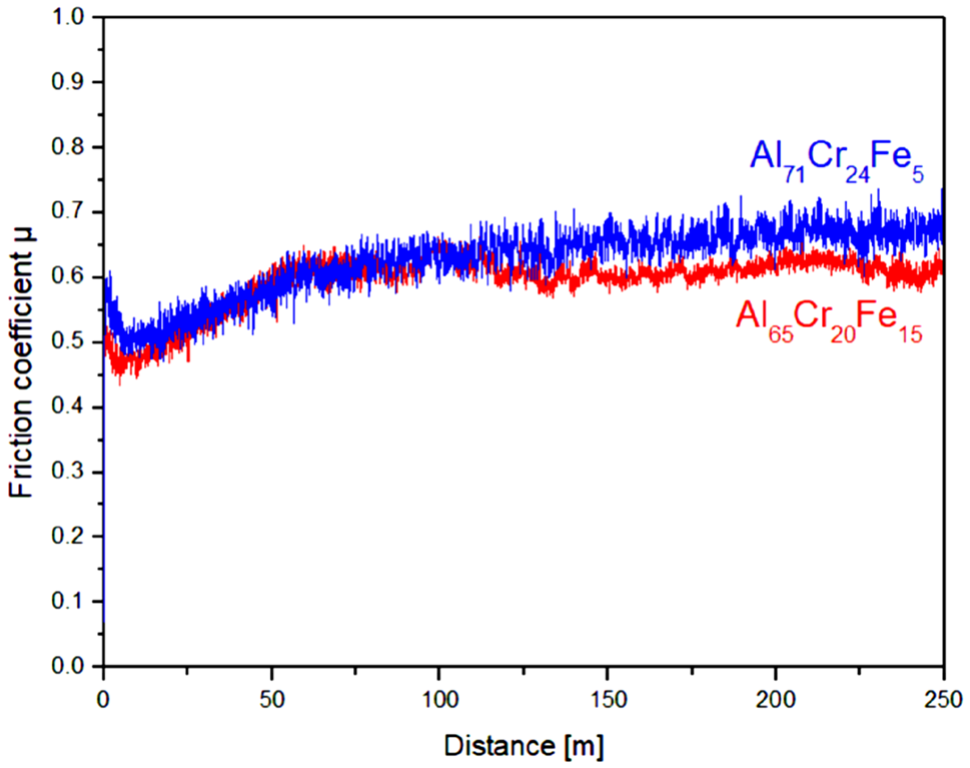
Pin-on-disc curves of Al_65_Cr_20_Fe_15_ and Al_71_Cr_24_Fe_5_ (ingots) as the relation of friction coefficient in the function of sliding distance.

Many researchers study the surface properties of complex metallic alloys because of the specific electronic structure associated with high symmetry clusters and unit cells made of thousands of atoms. Quasicrystals, which are included in the group of complex metallic alloys, are characterized by a low coefficient of friction and high wear resistance^2^. To describe abrasion resistance, tribological tests using the pin-on-disc method were performed. The test measurements with the parameters described in article^22^ were carried out, however, no clearly signs of wear were observed, due to the low linear speed 0.05 m/s and the distance 8 m as well as relatively low load (F_N_ = 2 N). We used the parameters described in^23^. Figure 6 presents a graph of the dependence of the friction coefficient on the distance, which was recorded during the pin-on-disc for Al_65_Cr_20_Fe_15_ and Al_71_Cr_24_Fe_5_ in the form of ingots. It could be observed that the friction coefficient decreased to values of approximately 0.46 (Al_65_Cr_20_Fe_15_) and 0.5 (Al_71_Cr_24_Fe_5_) in the initial stage of the study. The gradual increase was visible after a distance of 25 m, stabilization occurred, in which the mean value of the coefficient of friction was 0.6 for Al_71_Cr_24_Fe_5_ and 0.55 for Al_65_Cr_20_Fe_15_. Based on the measurements conducted, it is visible that the Al_65_Cr_20_Fe_15_ binary phase alloy was characterized by a lower friction coefficient compared to the Al_71_Cr_24_Fe_5_ multiphase alloy.

The results of pin-on-disc tests were also described in^24^, which compared the Al–Cu–Fe–Cr and Al–Cu–Fe alloys used for the coatings. It could be compared that for the chemical composition with the addition of chromium, the friction coefficient was similar to the results described in this article for Al_65_Cr_20_Fe_15_ and Al_71_Cr_24_Fe_5_ alloys (µ ≈ 0.6)^24^. Dubois et al.^25,26^ compared the results of tribological tests for CMA alloys with different chemical compositions. In the publication, the orthorhombic Al–Cr–Fe and γ-Al–Cr–Fe alloys show the values of the friction coefficient in the range of 0.5 ÷ 0.6. However, it should be noted that in publications^25,26^ the pin-on-disc studies were carried out under vacuum. The authors^25,26^ noted that the characteristic friction values are lower than in the air atmosphere. This is because the oxide layer has a significant influence on the measured value of the friction coefficient. Taking into account the fact that the alloys described in our work are binary and multiphase, it could be assumed that the wear resistance is similar to the single phase alloys^25,26^.

**Figure 7:**
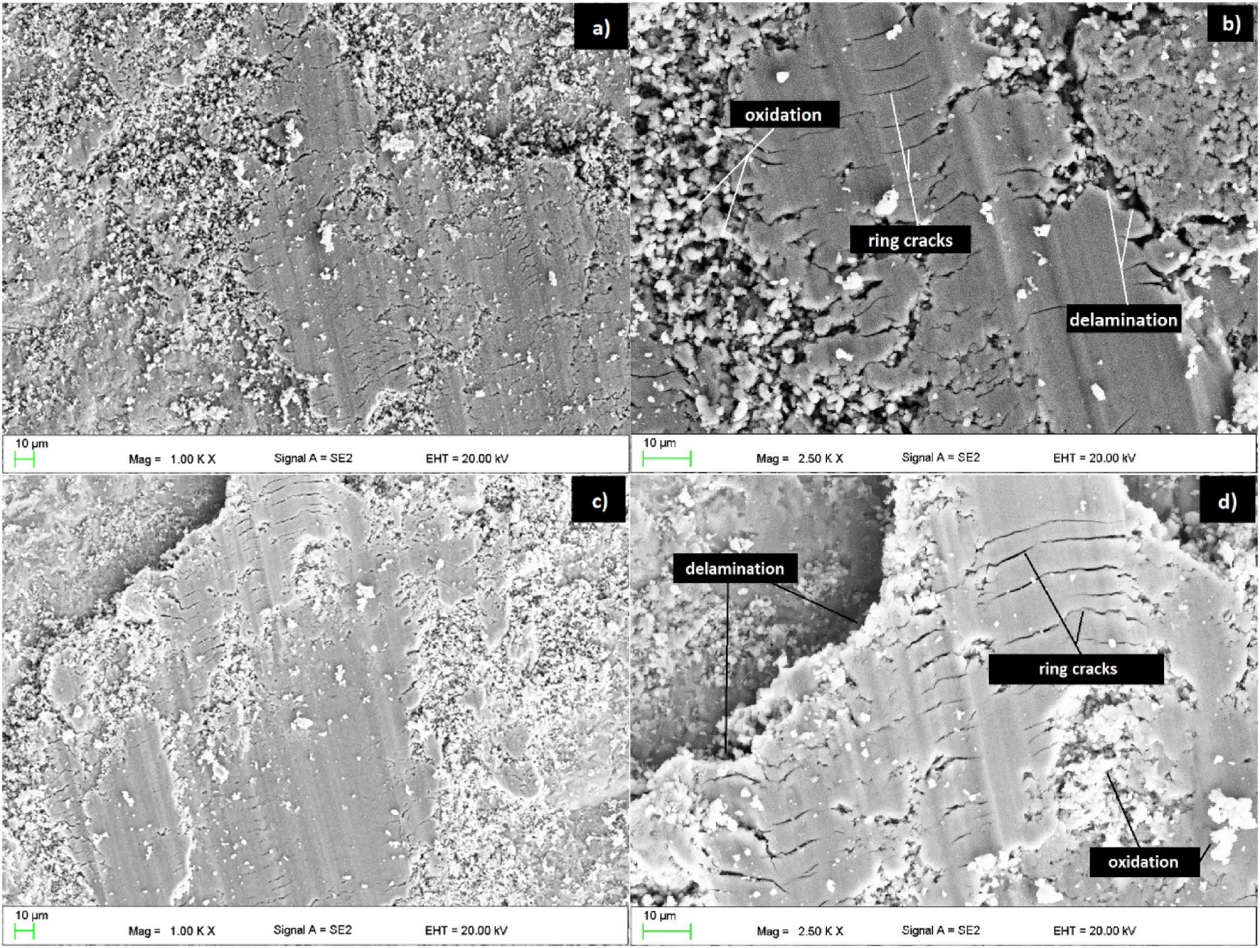
Surface morphology of the pin-on-disc friction track of (**a**, **b**) Al_65_Cr_20_Fe_15_, (**c**, **d**) Al_71_Cr_24_Fe_5_ alloys in a form of ingots.

Figure 7 shows the morphology of the wear tracks studied by SEM. It could be observed that three types of wear mechanisms dominated the trace of formation: plastic deformation, delamination, and oxidation. The identification of wear mechanisms was supported by the results described in paper^27^. Duckham et al.^28^ investigated the wear resistance of quasicrystalline Al–Pd–Mn and Al–Ni–Co alloys. Wear tracks were also observed after pin-on-disc tests using microscopic methods. The authors^28^ paid attention to the characteristic cracks that also appeared for the Al_65_Cr_20_Fe_15_ and Al_71_Cr_24_Fe_5_ alloys studied. This mechanism is called by^28^ as the ring cracks, characteristic of brittle materials, which indicates the maximum tensile stress. The article^28^ also describes the partial removal of the material, which is a delamination. The publications of Dubois et al.^25,26^ described the phenomenon of oxidation during pin-on-disc tests caused by the air atmosphere, which was observed using SEM in the form of oxide debris. Furthermore, wear track width measurements were carried out, the mean values of which were 1.23 (± 0.05) and 1.27 (± 0.07) for the Al_65_Cr_20_Fe_15_ and Al_71_Cr_24_Fe_5_ alloy, respectively. However, the literature lacks data on the width of the wear tracks for similar alloys and the same experimental conditions.

**Figure 8:**
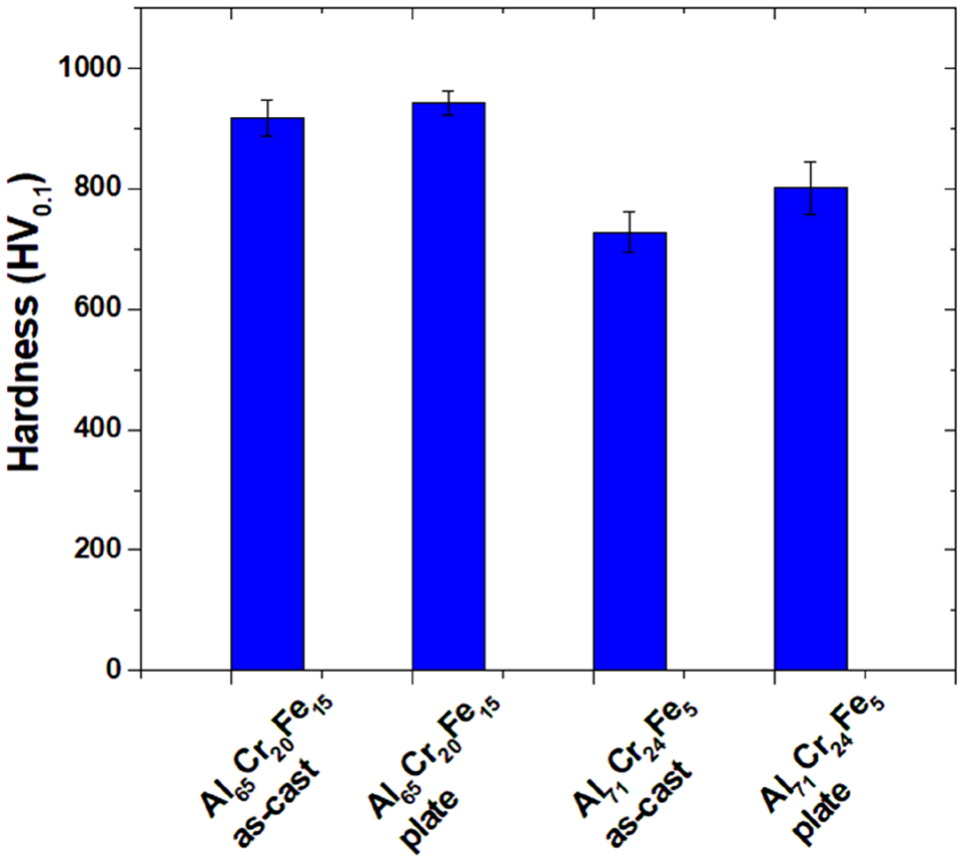
Comparison of hardness for Al_65_Cr_20_Fe_15_ and Al_71_Cr_24_Fe_5_ alloys in a form of ingots and plates.

The results of the average hardness measurements by the Vickers method together with the standard deviation for the Al_65_Cr_20_Fe_15_ and Al_71_Cr_24_Fe_5_ alloys in the form of ingots and plates are presented in the form of a bar graph in Fig. 8. Al_65_Cr_20_Fe_15_ in the form of ingots with a binary phase structure showed an average value of 917 (± 30) HV_0.1_, while the multiphase Al_71_Cr_24_Fe_5_ 728 (± 34) HV_0.1_. In the case of both chemical compositions, a clear effect of the application of the increased cooling rate of the liquid state was observed due to the values obtained of 943 (± 20) HV_0.1_ for Al_65_Cr_20_Fe_15_ and 802 (± 43) HV_0.1_ for Al_71_Cr_24_Fe_5_. The values obtained seem to be interesting due to the data in the comparison of the literature^25,26^ according to which the hardness for γ-Al–Cr–Fe is just over 700 HV. Another article^2^ presents the mean value of Vickers hardness for γ-Al_67.6_Cr_23.3_Fe_9.1_ 840 (± 50) HV.

**Figure 9:**
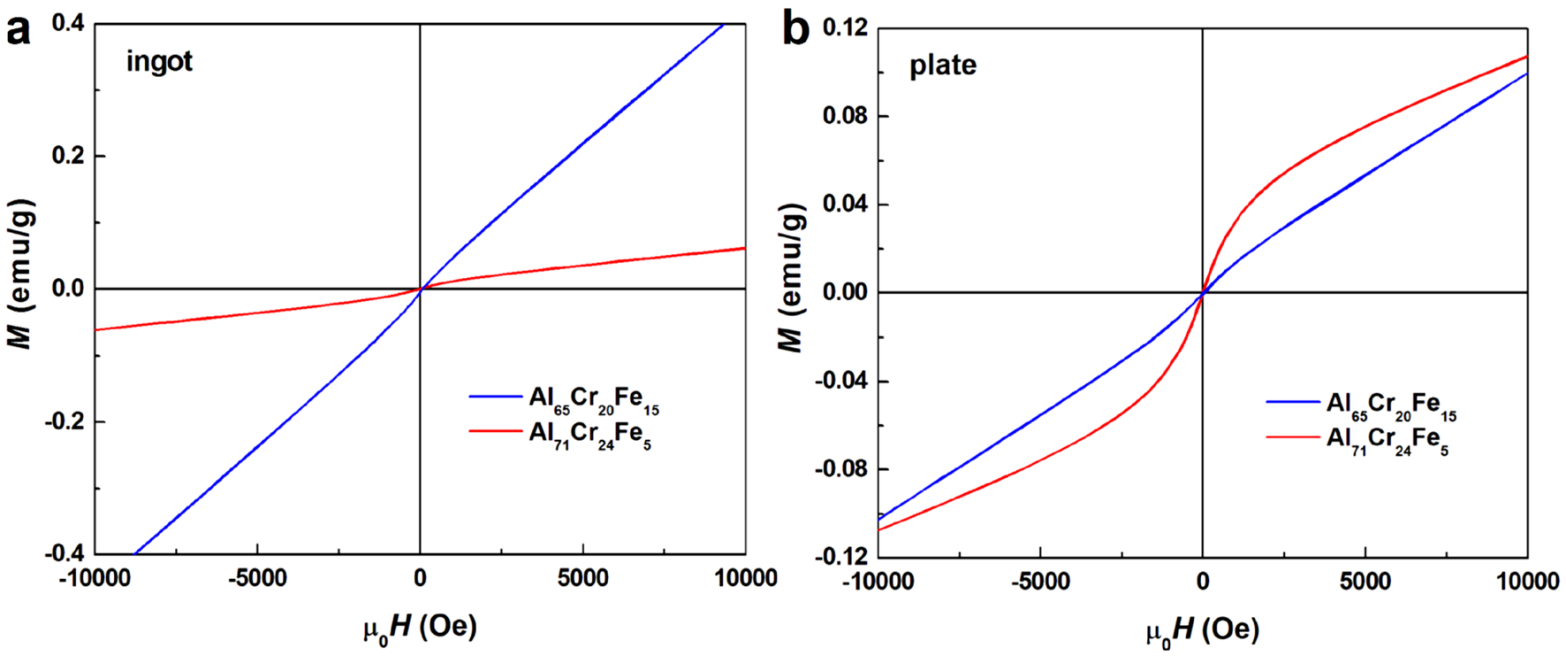
Magnetization (M) as a function of the magnetic field (µ_0_H) at room temperature for Al_65_Cr_20_Fe_15_ and Al_71_Cr_24_Fe_5_ alloys in a form of ingots (**a**) and plates (**b**).

According to Dubois^29^, quasicrystals and their approximants with good lattice excellence are characterized by diamagnetic properties in a wide range from ~ 50 K to the melting point. Among others, quasicrystalline Al-Cu-Fe alloys at the temperature of 2–300 K (− 271–27 ℃) showed diamagnetic properties^30^. According to the article^30^, the paramagnetic properties for Al–Cu–Fe alloys result from the participation of crystalline phases or structural defects. The Al_86_Cr_8_Fe_6_^31^, Al_61.3_Cr_31.1_Fe_7.6_^32^, Al_80_Cr_15_Fe_5_^33^ alloys were characterized by paramagnetic properties. According to Bihar et al.^32^, in the γ-AlCrFe approximant, the iron atoms are carriers of the magnetic moments. Therefore, as a result of the presence of two and many phases in the structure of the examined alloys, magnetic tests seem to be a significant supplement to the current state of knowledge. Especially since there still exists a small amount of data on the magnetic properties of structurally complex alloys. Changes in magnetization in a function of magnetic field for Al_65_Cr_20_Fe_15_ and Al_71_Cr_24_Fe_5_ in the form of ingots and plates were illustrated in Fig. 9. The values of saturation magnetization (*M*_*s*_) and coercivity (*H*_c_) were listed in Table 1. In numerous studies, the influence of the structure on the magnetic properties was observed. In the case of the Al_65_Cr_20_Fe_15_ alloy, the saturation magnetization was higher for the ingot form. The relationship was opposite and a lower value was noted for the ingot of the Al_71_Cr_24_Fe_5_ composition. The coercivity was several times higher for the plates in both chemical compositions. It may be related to changes in the structure under the influence of the cooling rate from the liquid state. In work^34^ for Fe-based alloys with nanocrystalline structure, changes in coercivity resulting from grain growth after annealing were observed. In this work, the opposite phenomenon was observed because increasing casting conditions lead to fragmentation of the structure. The alloys studied showed paramagnetic properties. On the basis of the obtained results, a decrease of magnetic properties is visible along with an increase in the cooling rate from the liquid state for the Al_65_Cr_20_Fe_15_ alloy and an increase for the Al_71_Cr_24_Fe_5_ alloy. Paramagnetic properties were also described in^2,33^ for the single phase Al_80_Cr_15_Fe_5_ SCAP-type alloy. Furthermore, polycrystalline, structurally complex Al_86_Cr_6_Fe_6_^31^ and Al_61.3_Cr_31.1_Fe_7.6_^32^ alloys were previously described in the literature as paramagnets. Based on the research conducted, it could be concluded that the presence of crystalline phases in the Al_65_Cr_20_Fe_15_ and Al_71_Cr_24_Fe_5_ alloys did not change the magnetic properties at room temperature.

**Table 1:** The magnetic properties of Al_65_Cr_20_Fe_15_ and Al_71_Cr_24_Fe_5_ determined by analysis of the magnetization changes as a function of magnetic field (*M*_*s*_, saturation magnetization; *H*_*c*_, coercivity).

Sample	Type	*M*_*s*_ (emu/g)	*H*_*c*_ (Oe)
Al_65_Cr_20_Fe_15_	Ingot	0.43	3.93
	Plate	0.09	27.69
Al_71_Cr_24_Fe_5_	Ingot	0.06	5.23
	Plate	0.11	21.85

The corrosion resistance of Al–Cr–Fe alloys was reported in the paper^20^. Electrochemical measurements of the open circuit potential as a function of time and potentiodynamic polarization curves were recorded in a 3.5% NaCl aqueous solution at a temperature of 25 °C. Electrochemical impedance spectroscopy tests were also carried out. The electrochemical parameters, such as *E*_OCP_, *E*_corr_, *R*_p_, and *j*_corr_ for studied Al-Cr-Fe alloys varied, which indicates differences in the corrosion mechanism. Among others, the Al_65_Cr_20_Fe_5_ alloy in the form of plate showed a corrosion potential closer to the positive values, although a higher polarization resistance was observed for the Al_71_Cr_24_Fe_5_ plate. Analysis of corrosion products is a useful tool to assess the corrosion behavior of materials; therefore, this article presents the results of the XPS analysis for Al_65_Cr_20_Fe_15_ and Al_71_Cr_24_Fe_5_ alloys in the form of plates after corrosion tests^35^.

**Figure 10:**
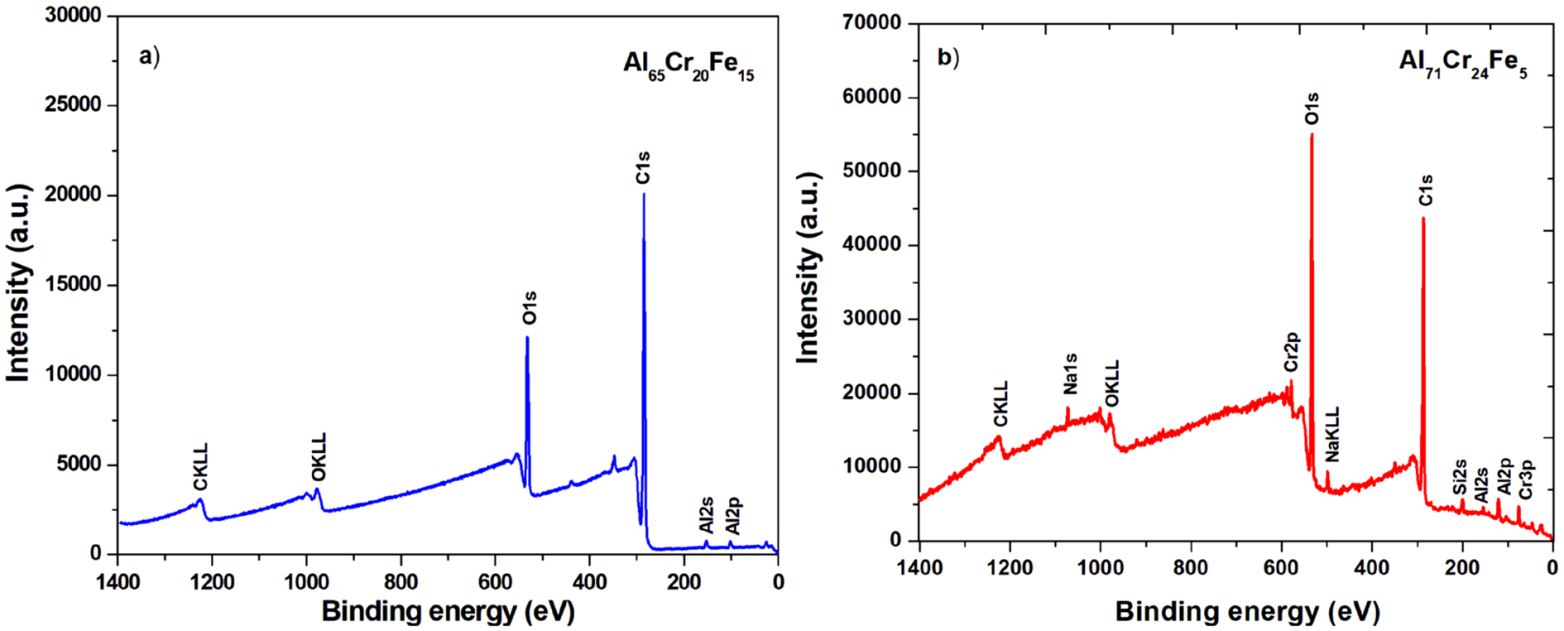
XPS survey spectra of Al_65_Cr_20_Fe_15_ (**a**) and Al_71_Cr_24_Fe_5_ (**b**) alloys in a form of plates after corrosion tests in 3.5% NaCl solution at 25 °C.

**Figure 11:**
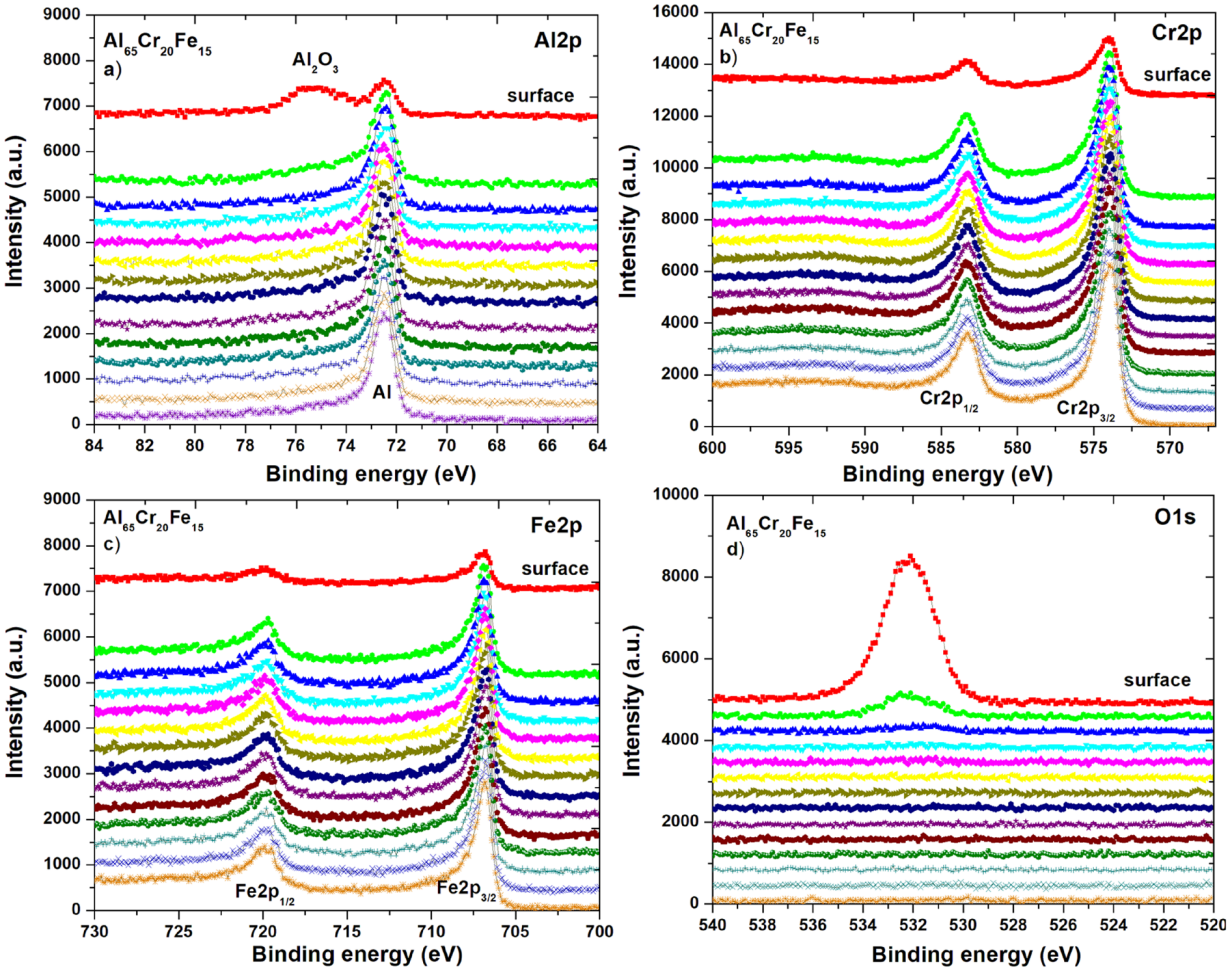
XPS core level lines of Al2*p* (**a**), Cr2*p* (**b**), Fe2*p* (**c**), O1*s* (**d**) of Al_65_Cr_20_Fe_15_ plate after corrosion tests in 3.5% NaCl solution at 25 °C.

**Figure 12:**
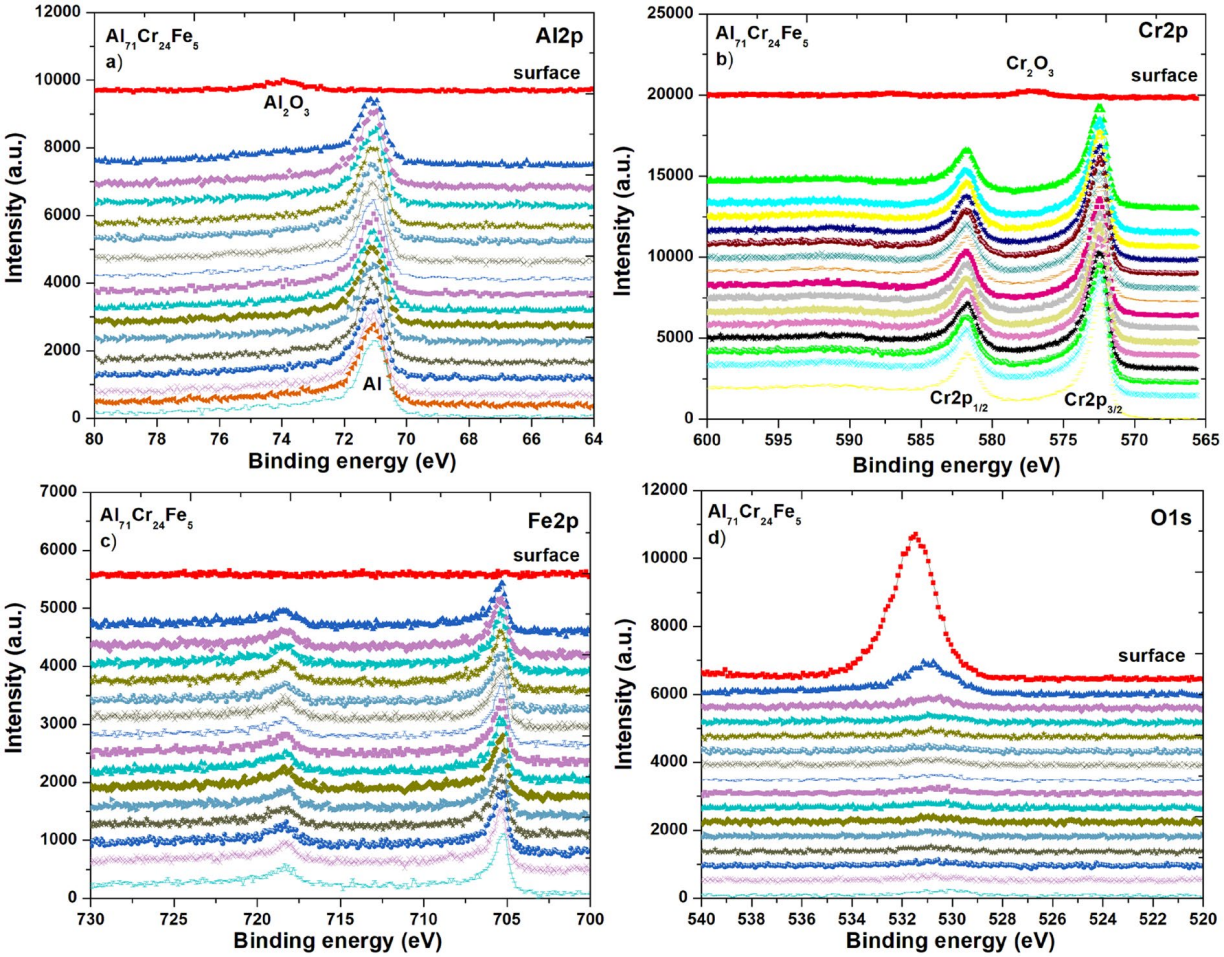
XPS core level lines of Al2*p* (**a**), Cr2*p* (**b**), Fe2*p* (**c**), O1*s* (**d**) of Al_71_Cr_24_Fe_5_ plate after corrosion tests in 3.5% NaCl solution at 25 °C.

**Figure 13:**
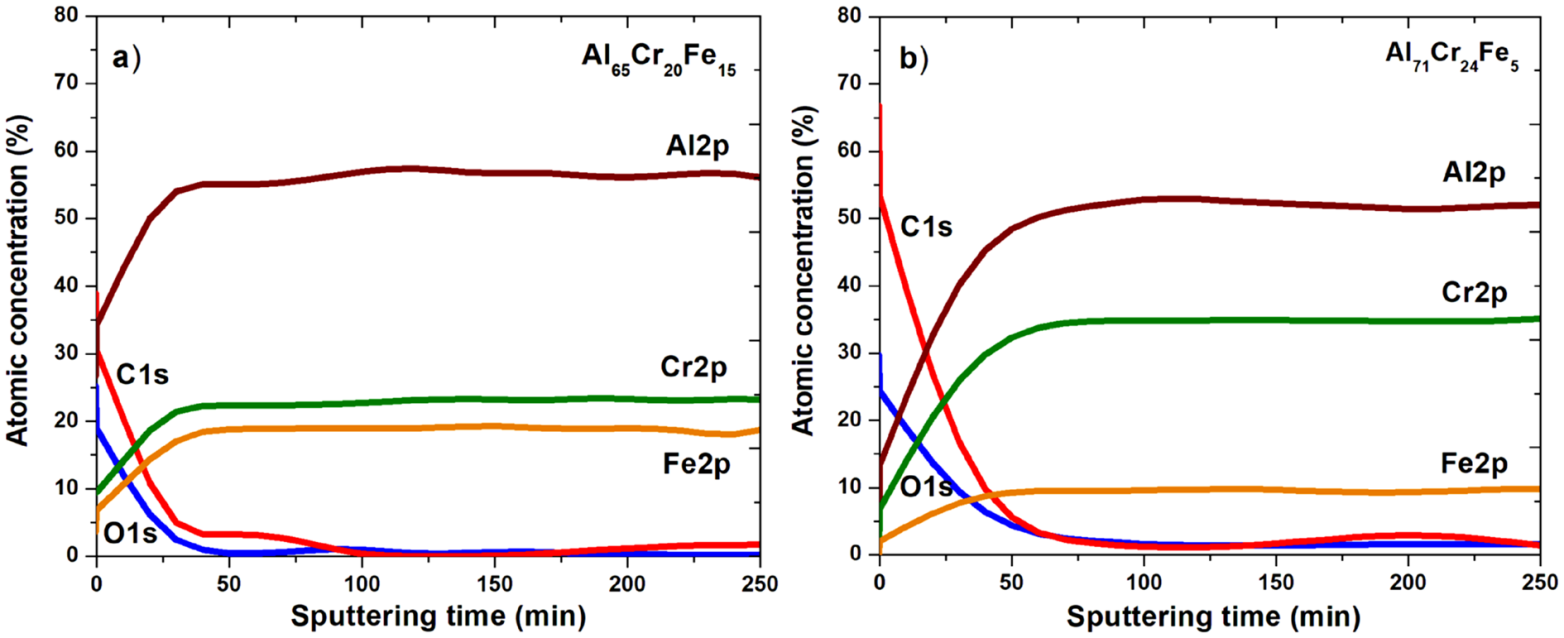
DP-XPS results for Al_65_Cr_20_Fe_15_ (**a**) and Al_71_Cr_24_Fe_5_ (**b**) alloys in a form of plates after corrosion tests in 3.5% NaCl solution at 25 °C.

The XPS survey spectra for the surface of Al_65_Cr_20_Fe_15_ (a) and Al_71_Cr_24_Fe_5_ (b) in the form of plates are presented in Fig. 10. The characteristic peaks (O1*s*, C1*s*, Al2*s*, Al2*p*, Cr2*p*, Cr3*p*) and the Auger spectrum (for O KLL and C KLL) were identified. High intensities relative to the binding energy of oxygen may indicate the formation of a passive layer in the tested plates. Figures 11 and 12 present the XPS core level lines of Al2*p*, Cr2*p*, Fe2*p*, O1s acquired during depth profile measurements for Al_65_Cr_20_Fe_15_ and Al_71_Cr_24_Fe_5_ plates, respectively. As one may notice at the surface, high binding energy Al2*p* and Cr2*p* peaks typical for oxides are evident. These results indicate the formation of a passive layer of Al_2_O_3_ and Cr_2_O_3_. Along with removing successive atomic layers by the argon beam, Al2*p* and Cr2*p* are typical for pure aluminium and chromium elements. It is worth noting that for both plate samples, the Fe2*p* line is typical for metallic iron with a spin–orbit splitting of about ΔE ≈ 12.8 eV. The XPS depth profiles for plates Al_65_Cr_20_Fe_15_ (a) and Al_71_Cr_24_Fe_5_ (b) are shown in Fig. 13. As the sputtering time and depth increased, the samples analyzed showed a significantly lower percentage atomic concentration of C1*s*, indicating the presence of carbon impurity usually accumulated on the surface. In the case of O1*s*, the same tendency can be noticed. Oxygen in the initial stage of sputtering may indicate the presence of oxygen, as a typical impurity on the surface that overlaps with oxygen formed by the passive layers. As successive atomic layers of the argon beam are removed and the depth of the tested material increases, the atomic concentrations of Al, Cr, and Fe are higher than at the surface.

According to data from the work^1^, the Al–Cr–Fe alloys should be characterized by good corrosion resistance, especially with high proportions of Al and Cr. These elements are passivating and allow the formation of a protective layer against further corrosion. Furthermore, Ott et al.^36^ studied the polycrystalline γ-Al_64.2_Cr_27.2_Fe_8.1_ alloy. Based on results^36^, it was found that the addition of chromium is necessary for the stabilization of the passive layer. Therefore, the Cr_2_O_3_ was identified for the Al_71_Cr_24_Fe_5_ alloy with a higher chromium content, which positively influences the corrosion resistance.

## Conclusions

- Structural studies using ND, SEM–EDX, and TEM methods confirmed the presence of two phases for the Al_65_Cr_20_Fe_15_ alloy and multiple phases for Al_71_Cr_24_Fe_5_. Both alloys were characterized by the presence of the structurally complex alloy phase—Al_65_Cr_27_Fe_8_. The Al_8_Cr_5_ phase with γ-brass structure was identified for Al_71_Cr_24_Fe_5_ alloy in a form of ingot and plate.
- The binary phase Al_65_Cr_20_Fe_15_ alloy showed a lower friction coefficient compared to the multiphase Al_71_Cr_24_Fe_5_. The values of the coefficient of friction were similar for the single-phase CMA alloys described in the literature when the air atmosphere during the pin-on-disc tests was altered.
- The beneficial effect of the applied cooling rate on hardness was demonstrated for both chemical compositions. The binary phase Al_65_Cr_20_Fe_5_ alloy was characterized by higher hardness values compared to the multiphase Al_71_Cr_24_Fe_5_ alloy and the single phase Al–Cr–Fe alloy described in the literature.
- The Al_65_Cr_20_Fe_15_ and Al_71_Cr_24_Fe_5_ alloys studied showed paramagnetic properties. The Al_71_Cr_24_Fe_5_ alloy with an increase in the cooling rate of the liquid state showed an increase of the magnetic values.
- The studied Al_65_Cr_20_Fe_15_ and Al_71_Cr_24_Fe_5_ alloys were characterized by the presence of passive oxide layers after electrochemical tests: Al_65_Cr_20_Fe_15_ (Al_2_O_3_) and Al_71_Cr_24_Fe_5_ (Al_2_O_3_ + Cr_2_O_3_). The higher intensity of oxides on the surface of Al_71_Cr_24_Fe_5_ alloy was recorded because of higher chromium content that stabilizes a passive layer.

## Data Availability

The data and material generated during and/or analyzed during the current study are available from the corresponding author on reasonable request.
